# Is de-escalation of treatment by omission of radiotherapy associated with fear of cancer recurrence in women with early breast cancer? An exploratory study

**DOI:** 10.1007/s10549-023-07039-2

**Published:** 2023-07-22

**Authors:** Lesley Stafford, Michelle Sinclair, Phyllis Butow, Janemary Hughes, Allan Park, Leslie Gilham, Allison Rose, G. Bruce Mann

**Affiliations:** 1 Familial Cancer Centre, Royal Melbourne Hospital, Melbourne, VIC Australia; 2 Melbourne School of Psychological Sciences, University of Melbourne, Victoria, Australia; 3 Department of Surgery, University of Melbourne, Victoria, Australia; 4 School of Psychology, University of Sydney, Sydney, NSW Australia; 5 The Breast Service, The Royal Melbourne and Royal Women’s Hospitals, Parkville, VIC Australia; 6 Breast Cancer Trials, Newcastle, NSW Australia; 7 Northwestern BreastScreen, The Royal Melbourne Hospital, Parkville, VIC Australia

**Keywords:** Breast cancer, Radiotherapy, Fear of cancer recurrence, Quality of life, Treatment de-escalation

## Abstract

**Purpose:**

Safe de-intensification of adjuvant radiotherapy (RT) for early breast cancer (BC) is currently under evaluation. Little is known about the patient experience of de-escalation or its association with fear of cancer recurrence (FCR), a key issue in survivorship. We conducted a cross-sectional study to explore this association.

**Methods:**

Psychometrically validated measures including the Fear of Cancer Recurrence Inventory-Short Form were completed by three groups of women with early BC: Women in the PROSPECT clinical trial who underwent pre-surgical MRI and omitted RT (A), women who underwent pre-surgical MRI and received RT (B); and women who received usual care (no MRI, received RT; C). Between group differences were analysed with non-parametric tests. A subset from each group participated in a semi-structured interview. These data (*n* = 44) were analysed with directed content analysis.

**Results:**

Questionnaires from 400 women were analysed. Significantly lower FCR was observed in Group A (*n* = 125) than in Group B (*n* = 102; *p* = .002) or Group C (*n* = 173; *p* = .001), and when participants were categorized by RT status (omitted RT *vs* received RT; *p* < *.001*). The proportion of women with normal FCR was significantly (*p* < .05) larger in Group A (62%) than in Group B (35%) or Group C (40%). Two qualitative themes emerged: ‘What I had was best’ and ‘Coping with FCR’.

**Conclusions:**

Omitting RT in the setting of the PROSPECT trial was not associated with higher FCR than receiving RT. Positive perceptions about tailored care, lower treatment burden, and trust in clinicians appear to be protective against FCR.

**Supplementary Information:**

The online version contains supplementary material available at 10.1007/s10549-023-07039-2.

## Introduction

A priority in breast cancer (BC) care is to achieve optimal outcomes while avoiding both over-treatment and under-treatment of the disease. Recent advances show a steady progression towards de-escalation of treatment in lower risk disease, where patients are carefully selected for the most appropriate treatment approach to minimize toxicities and maximize outcomes [1]. Among these outcomes, psychosocial wellbeing, and quality of life (QoL) are key considerations.

One QoL outcome of particular significance in the context of BC is fear of cancer recurrence (FCR), defined as “fear, worry or concern relating to the possibility that cancer will come back or progress” [2]. FCR can become a concern shortly after diagnosis or treatment, persists long after treatment completion [3] and remains relatively stable over the survivorship trajectory [3]. It is one of the most common psychological phenomena in BC survivors.

FCR severity is most frequently measured using a subscale of the Fear of Cancer Recurrence Inventory (FCRI-SF) [4, 5]. On this scale, scores ≥ 13 indicate possible clinical FCR and warrant additional investigation, while scores ≥ 22 indicate clinical FCR that needs specialized intervention [3]. Approximately 60–88% of women with BC score ≥ 13, and 16–22% score ≥ 22 [3]. Clinical FCR is characterized by constant, intrusive thoughts about cancer; interpretation of mild, unrelated symptoms as a sign of recurrence; a belief that cancer will return regardless of actual prognosis; and an inability to make longer-term plans due to cancer worry[2]. FCR is a common reason for seeking professional clinical support and is associated with psychological distress, poorer social and occupational functioning, and increased health care costs [6].

While the prevalence and impacts of FCR in women with BC are well known, understanding of the predictors of FCR is still evolving. Surprisingly, prognosis and more intensive treatment have not been clearly associated with FCR [7]. More specifically, the association between adjuvant radiotherapy (RT) and FCR is unclear. In BC, a weak positive association between RT and FCR has been reported, consistent with other cancers [8].

While clinical trials have shown that adjuvant RT decreases the risk of local recurrence after breast conserving surgery for early BC, most women are cured with surgery alone [9]. RT is associated with significant short-term and longer-term morbidity, is expensive, and prolongs treatment duration [10]. Consequently, defining a sufficiently low-risk population in whom RT can be safely de-escalated is desirable. Many previous and ongoing studies seek to achieve this aim. One such study is PROSPECT (Post-operative Radiotherapy Omission in Selected Patients with Early breast Cancer Trial, ANZ-1002), a prospective non-randomized cohort study where women with unequivocally unifocal early BC on magnetic resonance imaging (MRI) and favourable surgical pathology were treated with breast conserving surgery and adjuvant systemic therapy but without adjuvant RT. Of the 443 patients registered for PROSPECT, 201 were treated on study without RT. The primary outcome of PROSPECT was reported in 2022, showing a very low rate of recurrence [11].

The impact of treatment de-escalation by omission of RT on FCR has not been systematically studied. Among trials of post-operative RT in BC, only the PRIME (Post-operative Radiotherapy In Minimum-risk Elderly) trial [12] included measurement of FCR. PRIME included women older than 65 years with low-risk BC treated with breast conserving surgery and endocrine therapy, with or without RT. This study did not employ a validated inventory to measure FCR but relied on qualitative analysis of free-text responses to the question ‘how did breast cancer impact on your life?’ Results indicate that 15 months post-surgery, 15% of both groups of women commented on FCR but by 5 years, this proportion was much lower. Anxiety about clinical follow-up increased in the first 15 months for all women but was consistently higher among women in whom RT was omitted, suggesting that women who received RT may have felt additional ‘protection’ from RT which would mitigate against FCR.

Clearly any attempts to reduce the physical morbidity of oncologic treatment must not compromise women’s psychological health, and as such, closer scrutiny of the association between FCR and omission of RT is warranted. We undertook a cross-sectional, retrospective study of a large sample of women with early BC to explore the association between receipt of RT and FCR. Given that RT is the standard of care, we hypothesized that women who received RT would have lower levels of FCR than women for whom RT was omitted.

## Methods

### Recruitment procedure

Participants were women with early BC, diagnosed between 2011 and 2019 who were at least 12 months post-diagnosis, and had undergone breast conserving surgery with sentinel node biopsy and/or axillary dissection. Women were recruited from a large tertiary hospital breast service in metropolitan Melbourne, Australia.

Participants enrolled in PROSPECT (ANZ-1002) [11] were approached. Women assessed as eligible for de-escalation following local staging with MRI and surgery who omitted RT comprised Group A of our sample; women deemed ineligible for de-escalation post-MRI and surgery who underwent RT comprised Group B. A third cohort of usual care patients, Group C comprised patients who had not undergone MRI, underwent adjuvant RT, and were never approached for participation in PROSPECT but were approximately matched on age, tumour grade distribution, and tumour size to Groups A and B.

All women able to consent and participate in English were invited by letter or email to complete a study questionnaire, either online or on paper. Invitations were followed with a phone call 2 weeks later. Where contact was unsuccessful, a second invitation was sent. Women who agreed to participate by completing the questionnaire and providing written informed consent, could opt into a semi-structured interview. Women were selected for interview based on their scores on the FCRI-SF to provide a range of FCR experiences. Interviews continued until saturation (no new themes emerging from three consecutive interviews) was achieved. The study received ethics approval [HREC approval number: 2020.002].

## Measures

### Questionnaire

Study questionnaires collected data on educational and relationship status, language spoken at home, parity, medical comorbidities, and current or past treatment for anxiety or depression. Tumour characteristics, nodal stage, age, and time since diagnosis were collected from medical records.

Quantitative psychometric outcomes were assessed with robust, well-validated patient-reported outcome measures. *Fear of cancer recurrence* was assessed using the 9-item short-form (severity) subscale of the FCRI (FCRI-SF). Scores range from 0 to 36 and cut-offs are as described above [4, 5]. *Negative affectivity*, a general disposition to experience subjective distress [13], a potential confounder of FCR [14–16], was measured with the 10-item Neuroticism subscale of the International Personality Item Pool [17].

### Semi-structured interview

The semi-structured interview was drafted by the first author, an academic psycho-oncologist with extensive clinical and research experience in BC and it was then refined in consultation with the multidisciplinary author group including another expert psycho-oncologist, specialist breast surgeon, breast radiologist, breast care nurse, and consumer with lived experience of BC. Semi-structured interviews were conducted telephonically by an experienced psychologist, and explored experience of BC treatment and the presence, nature, and extent of FCR (see Table 1). Interviews were recorded, electronically transcribed, and checked for accuracy.

**Table 1:** Semi-structured interview questions

- Can you tell me about your treatment for breast cancer? - If applicable: How did you feel/what did you think about having the PROSPECT MRI? (e.g. reassuring, worrying) - How, if at all, do you think breast cancer surgery affected or continues to affect your quality of life? - If applicable: Having had radiotherapy, can you describe whether it affected you? (If affected, how were you affected during treatment, afterwards, and how are you affected now?) - How do you think your experience of/recovery from breast cancer might have been different if you had/not had (as applicable) radiotherapy? - To what extent do you think about cancer coming back (recurrence), in your breast or somewhere else in your body? - Can you tell me more about these thoughts – when they occur, what you think about, how you manage these thoughts? - Hypothetically, if you had/had not had (whichever applicable) radiotherapy, do you think that you would still think about cancer recurrence in the same way? Would you think about it more or less? Why? - If applicable: Do you think that having the MRI had any impact on your thoughts about cancer recurrence? How? - Is there anything else that you would like to add that you think we should know about the impact of your breast cancer treatment or your participation in PROSPECT on your thoughts about recurrence?	

### Analyses

Quantitative data were analysed using SPSS version 28. Between group differences were analysed with non-parametric tests. Qualitative analysis, using Nvivo 12 software, followed the directed content analysis approach [18] informed by the literature, researcher expertise, and the study focus i.e. experiences of FCR, the extent to which treatment may have contributed to FCR and how women coped with FCR. Commonalities across existing FCR theory [19] and participant responses were used to identify preliminary coding categories. Two authors independently coded 20% of transcripts and developed the coding framework and potential themes, resolving disparities through discussion. The remaining interviews were then coded. Theme meanings were refined, and illustrative quotes identified.

## Results

### Study participation

**Fig. 1:**
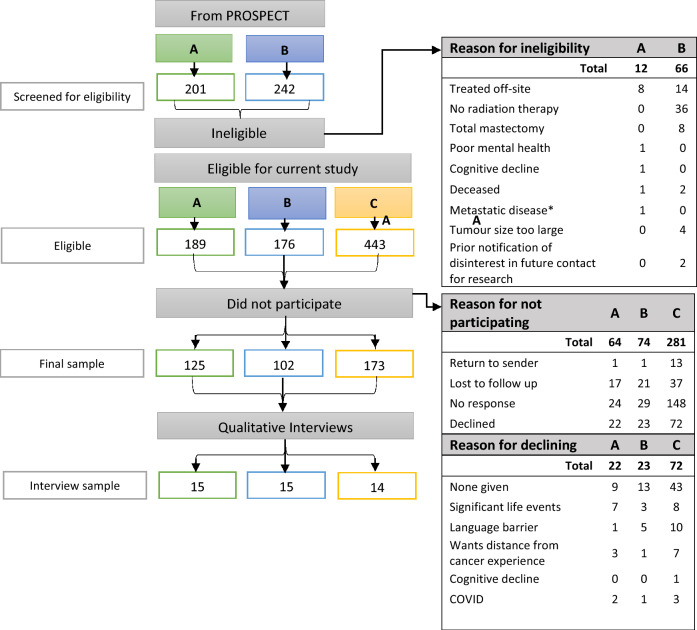
Study participation

Of eligible women who had participated in PROSPECT, 189 who had omitted RT (Group A) and 176 who had received RT (Group B) were contacted for participation. Of women who had RT in usual care, 443 were approached. The final sample included 400 women comprising 125, 102, and 173 in Groups A, B, and C, respectively. Of these participants, 79, 68, and 101 women from Groups A, B, and C, respectively, consented to participate in a semi-structured interview. Interviews were conducted with 15 participants each from Groups A and B and 14 from Group C. Details of study participation are shown in Fig. 1.

### Sample characteristics

Demographic and clinical characteristics of the sample are shown in Table 2. Most women were married (68.5%) and half had at least a secondary education (54.8%). Median age was 65 years, and median time since diagnosis was 4.4 years. There were no differences between groups in demographic and clinical groups except in tumour size. Participants in Group A had significantly smaller tumours (median = 10 mm) than their counterparts in Groups B and C (13 and 14 mm, respectively).

**Table 2:** Demographic and clinical characteristics of the sample

Characteristic	Group A *n* = 125		Group B *n* = 102		Group C *n* = 173		Total sample *N* = 400	
	Median	Range	Median	Range	Median	Range	Median	Range
Number of children^+^	2	0–6	2	0–7	2	0–6	2	0–7
Age (years)^+^	66	51–83	65	51–83	63	51–84	65	51–84
Neuroticism^+^	22	10–48	23	10–49	23	10–45	23	10–49
Highest Level of Education	N	%	N	%	N	%	N	%
Primary	4	3.2	4	3.9	6	3.5	14	3.5
Secondary	72	57.6	49	48.0	84	48.6	205	51.3
Trade/certificate	15	12	21	20.6	25	14.5	61	15.3
Undergraduate	15	12	12	11.8	22	12.7	49	12.3
Postgraduate	18	14.4	16	15.7	35	20.2	69	17.3
Relationship Status	N	%	N	%	N	%	N	%
Married/cohabiting	84	67.2	74	72.5	116	67.1	274	68.5
Single	8	6.4	5	4.9	12	6.9	25	6.3
Widowed	15	12	8	7.8	9	5.2	32	8.0
Divorced/separated	18	14.4	15	14.7	36	20.8	69	17.3
Mental health treatment	N	%	N	%	N	%	N	%
Currently	21	16.8	20	19.6	46	26.6	87	21.8
Previously	47	37.6	40	39.2	80	46.2	167	41.8
Chronic Medical conditions	N	%	N	%	N	%	N	%
Yes	57	45.6	53	52	91	52.6	201	50.3
Tumour size*^+^	10^a^	3–20	13^b^	4–30	14^b^	1–30	12	1–30
Months since diagnosis ^+^	49	14–109	51	13–118	56	12–120	53	12–120
Stage	N	%	N	%	N	%	N	%
1a	125	100	71	69.6	111	64.2	307	76.8
1b	0	0	7	6.9	14	8.1	21	5.3
2a	0	0	23	22.5	44	25.4	67	16.8
2b	0	0	1	1	4	2.3	5	1.3
Tumour Stage	N	%	N	%	N	%	N	%
T1a or T1b	73	58.4	31	30.4	53	30.6	157	39.3
T1c	52	41.6	60	58.8	82	47.4	194	48.5
T2	0	0	11	10.8	38	22	49	12.3
Nodal Stage	N	%	N	%	N	%	N	%
pN0	125	100	81	79.4	151	87.3	357	89.3
pN1mi	0	0	8	7.8	9	5.2	17	4.3
pN1	0	0	13	12.7	13	7.5	26	6.5
Nodal Status	N	%	N	%	N	%	N	%
Negative	125	100	81	79.4	151	87.3	357	89.3
Positive	0	0	21	20.6	22	12.7	43	10.8
Tumour Grade	N	%	N	%	N	%	N	%
1	62	49.6	30	29.4	39	22.5	131	32.8
2	56	44.8	56	54.9	93	53.8	205	51.3
3	7	5.6	16	15.7	37	21.4	60	15.0
Not Specified	0	0	0	0	4	2.3	4	1.0

Where percentages do not equal 100, this is due to missing data

^+^indicates differences between groups were assessed using non-parametric tests

^*^Difference between groups assessed as significant *p* < .001 as per independent samples Kruskal–Wallis test

^ab^Each subscript letter denotes a subset of group whose proportions do not differ significantly from each other; different subscript letters indicate the pairwise comparison found a significant difference between these groups (with Bonferroni correction < .05)

### Fear of cancer recurrence: quantitative outcomes

Comparisons of FCR across groups using Mann–Whitney U test are shown in Table 3. Women who omitted RT following MRI (Group A) had significantly lower FCR compared to those who had RT, whether compared to participants who: received RT after MRI (Group B), received RT in usual care (Group C), or a combination of all participants who received RT (Group B plus C). Undergoing MRI with RT was not associated with significant differences in FCR as demonstrated in the comparison between women in Groups B and C, both of whom received RT. The inclusion of MRI i.e. participation in the PROSPECT trial (Groups A and B) was associated with lower FCR when compared with FCR in Group C.

**Table 3:** FCR outcomes across groups

	Group A *n* = 125		Group B *n* = 102		Group C *n* = 173	
FCRI-SF	Median	Range	Median	Range	Median	Range
	11	0–32	14.5	0–33	14	0–32
FCR categories	N	%	N	%	N	%
< 13	77^a^	61.6	36^b^	35.3	69^b^	39.9
13–21	41^a^	32.8	56^b^	54.9	77^ab^	44.5
≥ 22	7^a^	5.6	10^ab^	9.8	27^b^	15.6

Comparisons						
	Test	A–C	B–C	AB–C	A–BC	A–B
		Z (*p*) r	Z (*p*) r	Z (*p*) r	Z (*p*) r	Z (*p*) r
FCRI-SF	MWU	3.24 (*p* = *.*001).188	NS	1.99 (*p* = *.047) .100*	3.62 (*p* < *.001*) .181	3.05 (*p* = .002*)* .202

*FCRI-SF* fear of cancer recurrence inventory-short form

< 13: FCR within normal range. ≥ 13: FCR warranting further investigation. ≥ 22: clinically significant fear of cancer recurrence. *MWU * Mann–Whitney U Test. Z: Standardized Mann–Whitney U Test statistic. *NS* not significant. r = effect size

^ab^ Each subscript letter denotes a subset of a group whose proportions do not differ significantly from each other in the 3 × 3 χ^2^ model, different subscript letters indicate significant differences between the proportions of these groups (with Bonferroni correction < .05)

A 3 × 3 χ^2^ test was used to compare categories of FCR severity between groups. As shown in Table 3, the proportions of women in each FCR category differed between the groups [X^2^ (4, *n* = 400) = 23.82, *p* < 0.001]. Specifically, the proportion of women who omitted RT (Group A) with normal levels of FCR (≤ 13) was significantly larger (62%) than the proportion of women with normal levels in the groups who received RT (Group B = 35%, Group C = 40%; *p* < 0.05). A significantly greater proportion of women in Group B (55%) than Group A (33%) scored 13–21 on the FCRI-SF (*p* < 0.05). This association between higher FCR and receipt of RT was shown again in the significantly larger proportion of women in Group C (16%) compared to Group A (6%) with scores ≥ 22 (*p* < 0.05). There were no other significant differences between groups.

A secondary analysis was conducted to eliminate any potential impact of disease severity on the analysis (see Appendix 1). All cases (*n* = 126) with any positive nodes, a Grade 3 tumour and tumour size > 20 mm were removed. The remaining sample comprised 274 women. There were no significant differences in age, time since diagnosis, parity, mental health treatment status or neuroticism between groups, and results were similar to the primary analysis. Women who omitted RT after MRI had significantly lower FCR than women who received RT after MRI (*p* = 0.008) and the combined group of women who received RT (*p* = 0.014). A significantly larger proportion of women in Group A (60%) had non-clinical levels of FCR (< 13) compared to Group B (34%), and a significantly smaller proportion of women in Group A (34%) had FCR levels warranting further clinical investigation (13–21) than in Group B (53%).

### Fear of cancer recurrence: qualitative outcomes

Qualitative analysis yielded two themes. The first theme, *‘What I had was best’* is inductive and describes how women managed their FCR by having trust in their treatment and faith in the medical advice they received. The second theme, *‘Coping with FCR’*, is deductive and comprises women’s descriptions of their FCR and coping as it related to their treatment. Theme descriptions and illustrative quotes for these themes are in Table 4.

**Table 4:** Illustrative quotes for the two themes ‘What I had was best’ and ‘*Coping with FCR’*

‘What I had was best’	Quotes
• Most women coped with FCR by believing their treatment was appropriate and necessary to treat the disease, even if treatment was difficult or unpleasant • Women placed trust in the recommendations of their doctors, the efficacy of treatments and the results of MRI imaging, which made treatment decisions easier, and minimized uncertainty and FCR • There was a general belief that the treatment plans were tailored. Women were reassured by learning that their cancer had been diagnosed early • Among women who did not have RT, there was a strong sense of gratitude for avoiding a treatment that was perceived as invasive (n = 11) • Some women who had MRI screening and RT (Group B) were disappointed at not being eligible for de-escalation (n = 6), and many expressed gratitude that they did not have chemotherapy. There was a general perception that more treatment equated to poorer prognosis. Some participants indicated that if they had received RT, their FCR could have been worse due to perception of a poorer prognosis and associated treatment burden (n = 7) • Omitting RT did not seem to be associated with increased FCR as these women believed they were at low risk of recurrence, trusted the information from their doctor, and believed they were receiving personalized treatment based on thorough assessment of their situation. These women also expressed the view that involvement in a trial came with the reassurance of additional monitoring • Trust in the recommended treatment plan based on medical advice, and for women who had MRI, imaging results, was so robust that 21 of the 29 interviewees who received RT suggested that they would have considered omitting RT if advised it was unnecessary • Some participants who underwent local staging with MRI reported that the experience was painful or distressing (n = 13); however, the discomfort was ‘worth it’ because the results were comprehensive and provided additional information beyond that of the mammogram and ultrasound. This bolstered their confidence in the recommended treatment and provided encouragement • Many women who received RT (Groups B and C) believed that it was preferable to have treated the cancer with RT as medically recommended and that omitting RT would have resulted in greater worry. Others reported that omitting RT would not have had an impact on their FCR (B = 6, C = 4) • The treatment burden, including ongoing side effects, were considered an acceptable compromise but some women noted that they were unprepared for RT side effects (B = 3, C = 6)	*“They explained to me that it was early days… we are lucky. We're in the early stages here…I had concerns about not having any follow up treatment initially, but I was guided by [*doctor’s name*] because well, he's qualified in his field. So I had confidence in him…and because I had an early cancer, I agreed to it [*to participate in PROSPECT*]… Should I have had radiation or should I not have? It's always light on my mind. But, you know, you have to be guided by the people that know. He is a qualified doctor. He knows what he's talking about”.* Participant A162 *“I found it really reassuring [the results of the MRI] in that it helped us to actually decide…the best way to go… [the doctor] wanted to go forward with radiation treatment… then I had to go forward with radiation and tamoxifen, but that's just, you know, good luck, [I’m] lucky to be able to have that quality of treatment”.* Participant B259 “*if I didn't have to have further treatment, I would have thought we've got it. And that's it… And when I think that chemo and radio[therapy], that must be really much worse. So if I just had a lumpectomy, and maybe these tablets…that would make it seem that it wasn't… as bad. You know… every extra thing you have, makes it seem like oh, it's a bit compounded, it's a bit worse, you have to have more treatment, whereas less treatment gives you the idea that it was, you know, it's gone. I don't have to worry as much”.* Participant B301 *“if the doctors and all the professional said you don't need radiotherapy, your sort of breast cancer doesn't need it. It's not going to make any difference to it coming back. Yes. I probably would have said, All right, well, I won't have it… You're totally in their hands. And you've got to just… put your trust in people, don't you?…”* Participant C838

‘Coping with FCR’	Quotes
• Levels of FCR varied within the sample and within each cohort. Many women described FCR as a constant presence in their lives, always in the back of their minds (A = 6, B = 7, C = 7) and that they took active steps to manage thoughts of recurrence • Worries about recurrence intensified in response to triggers including news of someone else’s cancer diagnosis or death, unusual physical sensations (e.g. pain in the breast), thoughts about treatment or taking medication, upcoming surveillance, and media reports of cancer • Women who received RT experienced triggers more intensely and spoke about triggering events more frequently and in greater detail. Indeed, women who omitted RT were more likely to comment that they did not have thoughts of recurrence or that these thoughts did not bother them (A = 9 vs B = 2, C = 2) • Women with FCR reported coping by pushing thoughts of recurrence out of their minds, distraction, and reassurance seeking. It was challenging for these women to speculate about what their experience of FCR may have been if they had undergone an alternative treatment pathway (omitting RT) • As an extension of this, some women had difficulty reconnecting with their treatment experience. Many women initially stated that RT had no or minimal impact on them (B = 11, C = 7); however, when directly questioned, they described how difficult RT had been for them at the time. They reported pushing the unpleasantness of RT out of their minds in a similar way to their thoughts of recurrence. When this strategy failed, reassurance was found through imaging and medical consultation, but some expressed anxiety about scans not detecting distant metastases (n = 5). Women on endocrine therapy reported this treatment as an additional source of reassurance	*“it's always there [fear of cancer recurrence.] And I just don't want to dwell on it. I think about it sometimes… when something triggers me…I try and keep myself busy…But I have found myself sort of avoiding it [thinking about cancer]”.* Participant C508 *“Actually, I don’t give it a thought during the year [cancer]… Don’t even concentrate on that side of things. Except when I look at the appointment book and I go, oh, it’s mammogram time again. And then…I still feel that I’m okay because I don’t have any tenderness. I don’t have any side effects… But it’s just when I have the mammogram and they reassure me…It’s been good every year and I feel like I’m on a good track, on the right track”.* Participant A15 *“Look, I don't think, I don’t dwell on it [how radiation impacted her]. So I don't think it really affects me now… I mean, the radiation towards the end, it was terrible. I was so burned. And I was so tired. It was really painful…., I try not to dwell on it… I obviously started getting tired, and then the burning would start. And towards the end, I just I just wanted it to be over. It was blood draining”.* Participant B336 *“It's constantly in my mind [fear of cancer recurrence]… I do worry now because even if I have an annual mammogram, it could recur in some other part of the body. And I've had two friends in the last 12 months pass away…. And each of them died after a recurrence of cancer…. I know very well that a mammogram is not going to detect recurrence in some other part of my body”.* Participant B329

## Discussion

Avoiding over-treatment and minimizing treatment-related physical morbidity in lower risk BC is of growing importance but the effect of de-escalation on mental health, including FCR, must also be carefully considered. We sought to explore this association using a combination of validated psychometric assessment and qualitative interviews.

Our findings from this exploratory study provide preliminary but novel data that in a select but large sample of women with early BC, those who omit adjuvant RT after pre-operative MRI do not experience *higher* FCR than their counterparts who subsequently undergo RT, as well as women in usual care who do not have MRI but have RT. This finding was consistent whether treating FCR as a dimensional variable or using recommended cut-offs. A secondary analysis in which women with any positive nodes, a Grade 3 tumour or tumour size > 20 mm were excluded, yielded the same result. The results are made more compelling by the lack of significant differences between groups in time since diagnosis, age of participants, mental health treatment status, and neuroticism.

There are several possible explanations for our findings. It may be that RT is associated with higher FCR due to prolonging the treatment experience, with side effects serving as a reminder of cancer and/or being interpreted as signs of recurrence [20]. Certainly, interview data showed that many women did experience toxicities, and expressed a need for more education prior to RT. RT recipients consequently had more triggers for FCR and reported higher levels of FCR than women who omitted RT. Indeed, many women who omitted RT described very positive treatment experiences and BC having a minimal impact on their lives.

Another plausible explanation is that adjuvant treatment may be perceived as indicating more serious illness. Women who omitted RT in this study expressed relief that their cancer was ‘caught early,’ and others who received RT noted that adjuvant RT ‘compounded’ their illness experience. Those deemed ineligible for de-escalation may have interpreted ineligibility as signalling a significantly worse prognosis. The way in which news of ineligibility for de-escalation was communicated to patients is unknown, but this too may have contributed to FCR if conveyed as indicative of a poorer prognosis.

Another potential contributing factor explaining lower FCR in the cohort who omitted RT is the close monitoring and personalized care from dedicated trial staff in PROSPECT. This is supported by the qualitative data. Further, although women from all three groups reported high levels of trust in the recommendations of their treating team, the additional prognostic information afforded by MRI may have been protective for FCR by providing even more reassurance. Women who omitted RT did not perceive RT omission as under-treatment, rather as appropriate treatment.

### Limitations and future research

Interpretation of these findings is limited by the cross-sectional, retrospective study design and recruitment from a single breast service. The groups studied were a priori clinically different in order to test the PROSPECT hypothesis. However, the absence of significant differences between the groups on age, time since diagnosis, neuroticism, and current or past mental health treatment is reassuring. It is possible that patients who declined participation in PROSPECT were more anxious and therefore disinterested in the option of treatment de-escalation. The exclusion of women not able to participate due to language barriers is recognized as a limitation.

There are several other psychological variables that could account for the differences reported here. Illness perceptions (personal ideas that patients have about their illness) may be important to consider. For instance, stronger beliefs about personal control over BC are related to fewer worries about whether cancer has been cured [21] and ideas about the chronicity, consequences, and emotional representation of BC have been associated with FCR [22]. Perceived risk of recurrence and appraisal of that risk are a key part of illness representations and are also associated with FCR [22, 23]. Future studies would benefit from including these variables. The way outcomes of investigations (e.g. pre-surgical MRI staging) and implications for treatment (e.g. eligibility for de-escalation) are communicated to patients and the meaning attributed to this information in terms of prognosis also warrants further scrutiny.

Nonetheless, the preliminary data presented here are novel and provide compelling grounds on which to include FCR in future studies of similar treatment de-escalation. If large-scale replication of PROSPECT confirms that omission of RT is associated with only a small rate of local recurrence in this select group, it may mean that QoL impacts, like FCR, become the deciding factor in determining whether or not to undergo RT.

### Clinical implications

Within the limits of this study, omitting RT in this setting does not appear to be associated with higher FCR, and this is reassuring for clinicians and patients attempting to limit treatment burden through de-escalation. Our findings may be of particular relevance to women ≥ 70 years with oestrogen receptor-positive, clinically node-negative T1 tumours for whom omission of RT is guideline-concordant. Further, providing clear communication, fostering trust in the patient-doctor and reassuring patients that their treatment plan is personalized may facilitate lower FCR.

## Conclusions

These findings provide preliminary but novel evidence that a select group of women with early BC who omit adjuvant RT after pre-operative MRI do not experience *higher* FCR than their counterparts who subsequently undergo RT, as well as women in usual care who do not have MRI but have RT. Future studies of de-escalation should include measurement of FCR and explore the role of illness perception variables and clinician communication in determining outcomes.

## Supplementary Information

Below is the link to the electronic supplementary material.

Supplementary file1 (DOCX 28 KB)

## Data Availability

The datasets generated during and/or analysed during the current study are available from the corresponding author on reasonable request.

## Abbreviations

BC: Breast cancer
FCR: Fear of Cancer Recurrence
FCRI-SF: Fear of Cancer Recurrence-Short Form
MRI: Magnetic Resonance Imaging
PRIME: Post-operative Radiotherapy In Minimum-risk Elderly
PROSPECT: Post-operative Radiotherapy Omission in Selected Patients with Early breast Cancer Trial
QoL: Quality of Life
