# Supplementary material for: Is de-escalation of treatment by omission of radiotherapy associated with fear of cancer recurrence in women with early breast cancer? An exploratory study
Source: Breast Cancer Res Treat. 2023 Jul 22;201(3):367–76. doi: 10.1007/s10549-023-07039-2 (PMC10460737; doi:10.1007/s10549-023-07039-2)
Supplement: Supplementary file 1 — Supplementary file1 (DOCX 28 KB) [file 10549_2023_7039_MOESM1_ESM.docx]

# Appendix A

*Table 1.*

Demographic and clinical characteristics of secondary analysis sample (n=274)

| **Characteristic** | **Group A**  **n=118** | | **Group B**  **n=62** | | **Group C**  **n=94** | | **Total sample**  **N=274** | |
| --- | --- | --- | --- | --- | --- | --- | --- | --- |
|  | **Median** | **Range** | **Median** | **Range** | **Median** | **Range** | **Median** | **Range** |
| Number of children^+^ | 2 | 0-6 | 2 | 0-7 | 2 | 0-6 | 2 | 0-7 |
| Age (years) ^+^ | 66 | 51-83 | 65 | 54-77 | 63.5 | 51-79 | 65 | 51-83 |
| Neuroticism^+^ | 21.5 | 10-48 | 22 | 10-49 | 24 | 10-43 | 23 | 10-49 |
| **Highest Level of Education** | **N** | **%** | **N** | **%** | **N** | **%** | **N** | **%** |
| Primary | 3 | 2.5 | 4 | 6.5 | 4 | 4.3 | 11 | 4.0 |
| Secondary | 67 | 56.8 | 28 | 45.2 | 50 | 53.2 | 145 | 52.9 |
| Trade/certificate | 14 | 11.9 | 16 | 25.8 | 14 | 14.9 | 44 | 16.1 |
| Undergraduate | 15 | 12.7 | 7 | 11.3 | 7 | 7.4 | 29 | 10.6 |
| Postgraduate | 18 | 15.3 | 7 | 11.3 | 18 | 19.1 | 43 | 15.7 |
| **Relationship Status** | **N** | **%** | **N** | **%** | **N** | **%** | **N** | **%** |
| Married/defacto | 79 | 66.9 | 44 | 71.0 | 70 | 74.5 | 193 | 70.4 |
| Single | 7 | 5.9 | 1 | 1.6 | 6 | 6.4 | 14 | 5.1 |
| Widowed | 15 | 12.7 | 6 | 9.7 | 3 | 3.2 | 24 | 8.8 |
| Divorced/separated | 17 | 14.4 | 11 | 17.7 | 15 | 16.0 | 43 | 15.7 |
| **Mental health treatment** | **N** | **%** | **N** | **%** | **N** | **%** | **N** | **%** |
| Currently | 19 | 16.1 | 13 | 21.0 | 25 | 26.6 | 57 | 20.8 |
| Previously | 46 | 39 | 21 | 33.9 | 42 | 44.7 | 109 | 39.8 |
| **Chronic Medical conditions** | **N** | **%** | **N** | **%** | **N** | **%** | **N** | **%** |
| Yes | 54 | 45.8 | 32 | 51.6 | 48 | 51.0 | 134 | 48.9 |
|  | **Median** | **Range** | **Median** | **Range** | **Median** | **Range** | **Median** | **Range** |
| Tumour size*^+^ | 10^a^ | 3-20 | 12^b^ | 4-20 | 11^ab^ | 1-20 | 11 | 1-20 |
| Months since diagnosis + | 49 | 14-109 | 49 | 13-118 | 58 | 12-120 | 53 | 12-120 |
| **Stage** | **N** | **%** | **N** | **%** | **N** | **%** | **N** | **%** |
| 1a | 118 | 100 | 62 | 100 | 93 | 98.9 | 273 | 99.6 |
| 1b | 0 | 0 | 0 | 0 | 1 | 1 | 1 | 0.4 |
| **Tumour Stage** | **N** | **%** | **N** | **%** | **N** | **%** | **N** | **%** |
| T1a or T1b | 69 | 58.5 | 22 | 35.5 | 40 | 42.6 | 131 | 47.8 |
| T1c | 49 | 41.5 | 40 | 64.5 | 54 | 57.4 | 143 | 52.2 |
| **Nodal Stage** | **N** | **%** | **N** | **%** | **N** | **%** | **N** | **%** |
| pN0 | 118 | 100 | 62 | 100 | 94 | 100 | 274 | 100 |
| **Nodal Status** | **N** | **%** | **N** | **%** | **N** | **%** | **N** | **%** |
| Negative | 118 | 100 | 62 | 100 | 94 | 100 | 274 | 100 |
| **Tumour Grade** | **N** | **%** | **N** | **%** | **N** | **%** | **N** | **%** |
| 1 | 62 | 52.5 | 23 | 37.1 | 30 | 31.9 | 115 | 42 |
| 2 | 56 | 47.5 | 39 | 62.9 | 61 | 64.9 | 156 | 56.9 |
| Not Specified | 0 | 0 | 0 | 0 | 3 | 3.2 | 3 | 1.1 |

Note: Where percentages do not equal 100, this is due to missing data

^+^ indicates differences between groups were assessed using non-parametric tests

* Difference between groups assessed as significant *p*<.05 as per independent samples Kruskal-Wallis test

^ab^ Each subscript letter denotes a subset of group whose proportions do not differ significantly from each other; different subscript letters indicate the pairwise comparison found a significant difference between these groups after Bonferroni correction (*p*<.05).

*Table 2.*

FCR outcomes across groups

|  | **Group A**  **n=118** | | | | **Group B**  **n=62** | | | | **Group C**  **n=94** | |
| --- | --- | --- | --- | --- | --- | --- | --- | --- | --- | --- |
|  | **Median** | | **Range** | | **Median** | | **Range** | | **Median** | **Range** |
| **FCRI-SF** | 11 | | 0-32 | | 14.5 | | 0-33 | | 13 | 1-32 |
| **FCR categories** | **N** | | **%** | | **N** | | **%** | | **N** | **%** |
| **<13** | 71^a^ | | 60.2 | | 21^b^ | | 33.9 | | 44^a,b^ | 46.8 |
| **13-21** | 40^a^ | | 33.9 | | 33^b^ | | 53.2 | | 41^a,b^ | 43.6 |
| **≥22** | 7^a^ | | 5.9 | | 8^a^ | | 12.9 | | 9^a^ | 9.6 |
| **Comparisons** | | | | | | | | | | |
|  | | **Test** | **A-C** | **B-C** | | **AB-C** | | **A-BC** | | **A-B** |
|  | |  | **Z (*p*) r** | **Z (*p*) r** | | **Z (*p*) r** | | **Z (*p*) r** | | **Z (*p*) r** |
| **FCRI-SF** | | **MWU** | NS | NS | | NS | | 2.45 (*p=*.014) .15 | | 2.65 (*p*=.008*)* .20 |

FCRI-SF: Fear of Cancer Recurrence Inventory- Short Form. <13: FCR within normal range. 13-21: FCR warranting further investigation. ≥22: clinically significant fear of cancer recurrence. MWU: Mann-Whitney U Test. Z: Standardised Mann-Whitney U Test statistic. NS: Not significant. r = effect size

^ab^ Each subscript letter denotes a subset of a group whose proportions do not differ significantly from each other in the 3x3 Chi-Square model, different subscript letters indicate significant differences between the proportions of these groups (with Bonferroni correction <.05).
